# Bridging gene therapy and next-generation vaccine technologies

**DOI:** 10.1038/s41434-024-00502-9

**Published:** 2024-11-18

**Authors:** Kristie Bloom, Abdullah Ely, Mohube Betty Maepa, Patrick Arbuthnot

**Affiliations:** Wits/SAMRC Antiviral Gene Therapy Research Unit, Infectious Diseases and Oncology Research Institute (IDORI), Faculty of Health Sciences, University of the Witwatersrand, Johannesburg, South Africa

**Keywords:** Infectious diseases, Respiratory tract diseases

## Contributions of the COVID-19 pandemic to gene-based vaccination

Dealing with the serious problems of COVID-19 has propelled vaccine research and the importance of pandemic preparedness to the fore. Current efforts to improve vaccine technologies and manufacturing capabilities are unprecedented. Global attention had concentrated on vaccination as a means of achieving herd immunity to prevent transmission and avoid serious complications of SARS coronavirus-2 (SARS-CoV-2) infection. With over forty COVID-19 vaccines receiving authorization, the focus has shifted towards refining public health responses and ensuring vaccine equity in preparation for the next pandemic. The concept that ‘No one is safe until we are all safe’ emphasizes the need to ensure vaccination campaigns rapidly and efficiently reach all populations in the world [1]. It is therefore problematic that COVID-19 vaccination rates were poor in Low and Middle Income Countries (LMICs) [2]. As of November 2021, it was estimated that less than 7% of Africans had been fully vaccinated against SARS-CoV-2. In addition to driving the pandemic, poor vaccine coverage contributes to emergence of variants of concern [2, 3]. The problem is further exacerbated by the high prevalence of HIV-1 infection in LMICs, which is associated with higher mortality from COVID-19. Furthermore, complications of HIV-1 infection contribute to prolonged SARS-CoV-2 infection, greater risk of emerging variants, spread and evasion of immunity. Availability of robust vaccination technology is thus vital to address a serious problem that is facing the world.

**Fig. 1:**
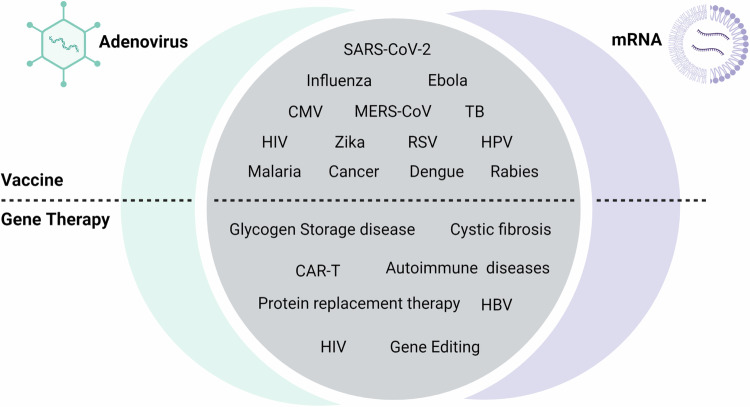
Examples of Adenovirus and mRNA vaccines and gene therapies in preclinical development. Parallels between Ad and mRNA platforms can be seen in both prophylactic and therapeutic vaccine development as well as for gene therapy (selected examples shown). CAR-T chimeric antigen receptor T-cell, CMV cytomegalovirus, HBV hepatitis B virus, HIV human immunodeficiency virus, HPV human papillomavirus, MERS-CoV Middle East respiratory syndrome coronavirus, RSV respiratory syncytial Virus; SARS-CoV-2: severe acute respiratory syndrome coronavirus 2, TB tuberculosis. Image created with BioRender.com/i23g678.

There are essentially two main types of vaccines: gene- and protein-based [4]. Gene-based vaccines, including attenuated viruses, recombinant viral vectors or mRNA-containing formulations, are typically designed to encode immunogens without mutating host DNA. Gene therapy contributed significantly to early development of gene-based vaccines. Interestingly the converse is also true in that rapid developments in the field of vaccinology have advanced gene therapy. Moreover, there is considerable overlap between advancing gene therapy and gene-based vaccines for disease management (Fig. 1). Developing gene-based vaccines is informed by uncomplicated rational design principles that use readily available sequence information. Sequences typically encode pathogen-derived immunogens, such as the Spike protein of SARS-CoV-2. To be efficient, vaccines should also possess adjuvant properties that stimulate the innate immune response to augment adaptive humoral and T cell immunity. However, these properties need to be balanced because strong activation of type I interferon responses and release of pro-inflammatory cytokines may cause systemic toxicity with attenuated vaccine efficacy. mRNA delivered in lipid nanoparticle (LNP) formulations usually activate Toll-Like Receptor 3 (TLR3), TLR7 and the inflammasome to induce a type I interferon (IFN) responses. Reduced binding of mRNA to TLRs and activating an innate immune response is effected by incorporation of modified nucleotides and elimination of contaminants, such as double-stranded RNA. Adenoviruses (Ads) have an inherent ability to target antigen-presenting dendritic cells and engage with TLR9 to activate a type I IFN response. Intracellular production of antigens combined with induction of innate responses by mRNA-containing LNPs and Ads contribute to efficient T cell priming, then differentiation into effector and memory cells. The adjuvant properties of mRNA vaccines are also likely to differ depending on lipid composition, mRNA modifications, and the mRNA-lipid combination.

Procedures for propagating viral vectors, such as recombinant Ads, or formulating mRNA in LNPs are generic and amenable to large scale platform manufacturing. Moreover, the technology has flexibility with respect to sequence alteration to adapt to changed viral targets. This is ideal for rapid vaccine development and is a reason for the prominence of mRNA LNPs and Ads in the response to the COVID-19 pandemic. Versatility of mRNA vaccination technology has been recognized by the WHO and led to advancing a hub and spoke program that is intended to build and disseminate pandemic preparedness capacity in LMICs [5].

## In vitro transcribed mRNA

Since the discovery of mRNA and elucidation of its critical role in gene expression, impetus has been given to exploring therapeutic and prophylactic uses of this molecule. Early experiments in the 1980s demonstrated feasibility of producing mRNA in vitro [6]. This first study reported expression of an encoded protein upon delivery of the mRNA to eukaryotic cells. Essential components of functional in vitro transcribed mRNA are: a 5’ cap, 5’ untranslated region (UTR), an open reading frame (ORF) and 3’ UTR with polyA tail. Better capping methods, use of cap analogs, incorporation of modified nucleotides, sequence optimization of UTRs, the ORF and polyA tailing have enabled significant improvement of synthetic mRNA technologies (reviewed in [7]). Valuable features of in vitro transcribed mRNA for clinical use include (i) ease of scale-up, (ii) good dose regulation because of a short half-life, (iii) low risk of recombination with host genomic DNA, and (iv) compatibility with highly efficient non-viral vectors.

Successful therapeutic or prophylactic use of in vitro transcribed mRNA is dependent on vectors to deliver mRNA to the cytoplasm of target cells. Essential functional requirements of these vectors are protection of the mRNA from degradation by nucleases, targeting intended cells and ensuring mRNA escapes from endosomes to the cytoplasm [8]. LNPs have proved to be effective and currently are popular mRNA vectors. Chemical synthesis of LNP components and convenient formulation procedures make them amenable to large scale accredited manufacturing. Utility of in vitro transcribed mRNAs has been highlighted by their rapid development in response to the COVID-19 pandemic. mRNA vaccines from Pfizer/BioNTech and Moderna, comprising lipid nanoparticles containing mRNA encoding the SARS-CoV-2 Spike protein, received rapid emergency approval and ultimately full approval from the FDA [9]. The vaccines induced good immune responses to SARS-CoV-2 and protected 90-95% of individuals against COVID-19 [10–12]. Several iterations have since received authorization, including monovalent and/or bivalent mRNA vaccine formulations encoding SARS-CoV-2 Spike proteins derived from Omicron variants (BA.1, BA.4/5 and XBB.1.5). This success has reignited the mRNA vaccine space, with most new drugs directed towards infectious diseases.

Although progress with mRNA technology has been impressive, the need for maintaining the vaccines at low temperatures is challenging. This is particularly problematic in resource-constrained settings. Use of lyophilized mRNA vaccine formulations that can be maintained at ambient temperature, then reconstituted immediately prior to administration, shows promise for improving stability [13]. Needle-free devices, including dissolving microneedle patches, have recently gained impetus as an alternative means of delivering mRNA vaccines [14, 15]. This technology aims to improve thermostability and simplify vaccine administration, as patches can be self-applied.

A complementary group of synthetic mRNAs, known as self-amplifying RNAs (saRNAs), incorporate sequences encoding non-structural alphavirus-derived elements. When introduced into cells, the saRNAs function as replicons and are amplified in situ. The longer mRNA half-life and enhanced immunogen expression are useful properties that reduce dose requirements compared to conventional mRNA. Several saRNA vaccine candidates have been developed to prevent infectious disease such as influenza, HIV, malaria, rabies, Zika, Ebola, Chikungunya, Tuberculosis, and Dengue Fever. Prior to the COVID-19 pandemic, few saRNA drug products had been trialed in humans despite encouraging pre-clinical studies. This landscape has changed, with over 40 active clinical trials currently underway for saRNA vaccines targeting infectious diseases (primarily SARS-CoV-2 and influenza) or cancers. Last year ARCT-154, a COVID-19 saRNA vaccine developed by CLS and Arcturus, received regulatory approval in Japan following promising clinical trial data [16]. This important milestone will help promote the clinical expansion of saRNA drug products as well as build the regulatory framework required for licensing these comparatively new drugs. However idiosyncratic RNA design and/or lipid formulations are likely to alter the immunogenicity profiles of saRNA vaccines. Results from the LNP-nCoVsaRNA phase 1 clinical trial (ISRCTN17072692) showed that although the LNP-formulated saRNA was deemed safe, sub-optimal anti-SARS-CoV-2 immune responses precluded phase 3 trials [17]. Conversely, results from the ARCT-021 phase 1/2 trial (NCT04480957) suggested high rates of seroconversion, even in the cohorts receiving a single dose [18]. Interestingly both studies noted that a longer prime-boost interval may be needed to improve immunogenicity of the second dose, to allow for maturation of the antibody response and better seroconversion, but further investigations are important to identify key design and dosing attributes that may enhance vaccine efficacy.

## Convergence with mRNA gene therapies

The successful translation of mRNA vaccines for clinical use was a significant development and is a stimulus for other applications of mRNA technology, especially therapeutic use of in vitro transcribed mRNA. This topic is being enthusiastically developed and offers a novel modality for combatting inherited genetic diseases, primarily as protein replacement or enhanced cell therapies, as well as intractable infectious diseases such as chronic hepatitis B (reviewed in [19]). Several mRNA-based protein replacement therapies are in early clinical trials for the treatment of cystic fibrosis (NCT06237335, NCT05668741, NCT03375047, NCT05712538), primary ciliary dyskinesia (NCT05737485), ornithine transcarbamylase deficiency (NCT06488313), familial hypercholesterolemia (NCT06458010), propionic acidemia (NCT05130437), methylmalonic acidemia (NCT05295433), chronic heart failure (NCT05659264), glycogen storage diseases (NCT04990388, NCT05095727), and phenylketonuria (NCT06147856). CRISPR-based therapies for transthyretin amyloidosis (NCT06539208) and hereditary angioedema (NCT05120830) make use lipid nanoparticles formulated with sgRNA and mRNA to achieve gene editing in vivo. Interestingly, mRNA therapies for cystic fibrosis and asthma (HWID59046) have been adapted to inhaled formulations, allowing cell targeted delivery. The dynamic and vast array of preclinical mRNA therapy programs currently underway suggests that we will see many new therapies enter clinical evaluation, particularly for neurological diseases and muscular dystrophy.

Currently, saRNA-based gene therapy research is largely limited to immunotherapy. However, given the diverse range of non-replicating mRNA gene therapies in development, it is likely that the technology will be expanded beyond application to vaccines. Concerns about undesired innate immune activation with suppression of translation may be delaying saRNA use. Including sequences encoding proteins that inhibit innate immunity in cis may reduce immunity to synthetic saRNA [20]. Strategies employing drug-inducible translation-controlling molecular switches are also being investigated [21]. Incorporating riboswitches along with microRNA target sites may help fine-tune saRNA pharmacokinetics by regulating translation and improving tissue-specific expression. These additional controls are likely to improve the overall safety of saRNA-based gene therapy products.

## Adenoviral vectors as gene therapy and vaccine development tools

First isolated in 1953, Ads are non-enveloped viruses with an icosahedral capsid that encases a double stranded DNA genome. The well-established biology and identification of over sixty Ad serotypes set the foundation for developing a highly versatile component of the gene therapy and vaccinology toolbox. Recombinant Ads are typically rendered replication-defective by removing sequences that are essential for their replication (e.g., E1) and replacing these elements with components encoding immunogenic or therapeutic proteins. The E3 region is also usually deleted in recombinant Ads to reduce immunostimulatory effects and increase packaging capacity [22]. Properties of Ads that have made them popular vectors include mild clinical features caused by transmission of the wildtype virus to immunocompetent individuals, large transgene capacity and good transduction efficiency.

Recombinant Ads induce strong innate and adaptive immune responses, which may be useful or unfavorable for vaccine development. Innate immune stimulation by Ads may cause severe toxicity and was dramatically demonstrated by the death of a clinical trial subject following systemic administration of a high dose of recombinant Ads for treatment of ornithine transcarbamylase deficiency [23]. After this fatality, Ads fell out of favor for gene therapy. However, induction of mild innate immune responses following low dose local injection may be useful as an intrinsic adjuvant. Adaptive immunity to the Ads themselves may be problematic. Immune responses to community-acquired Ad infections and vectors may diminish transgene delivery efficiency, especially when boosters are required. To overcome these problems, zoonotic Ads or viruses with low seroprevalence have been adapted as vectors [24]. For example the ChAdOx1 vaccine of AstraZeneca was developed from a chimpanzee Ad, and Johnson & Johnson (J&J) engineered the rare Ad26 to be used as a vector. Within a year after discovery of SARS-CoV-2, ten Ad-based SARS-CoV-2 vaccines were in clinical development and four were given approval for emergency use [24]. However, these vaccines have not gained popularity because of the serious, albeit rare, blood clotting disorders associated with their use [25–27]. The better safety profile of mRNA vaccines has led the US Centers for Disease Control and Prevention (CDC) to limit recommendations of Ad-based vaccines to specific indications, such as adults who refuse or cannot receive mRNA vaccines for medical reasons [28].

Several other Ad-based vaccines have been developed and are performing well in clinical trial. These have been aimed at neutralizing infection with Ebola virus, Influenza virus, *Mycobacterium tuberculosis* and *Plasmodium falciparum*. Unfortunately, Ad vaccination against HIV-1 has been unsuccessful. Problems caused by Ad-mediated immune stimulation were highlighted by a randomized controlled trial to compare preventative efficacy of a placebo and candidate HIV-1 vaccine comprising a recombinant Ad5 vector [29]. The vector expressed the HIV-1 *gag*, *pol* and *nef* genes and was used to assess prevention of HIV-1 infection. Pre-existing immunity to Ad5 was higher among vaccinated individuals and there was increased risk of HIV-1 infection in this group. It was thought that activation of HIV-1 antigen presenting cells by the vaccine supported early spread of the virus.

## Discussion and prospects

Vaccine research carried out in response to COVID-19 culminated in impressively efficient progress of mRNA- and Ad-based vaccines. However, the challenges of roll out to all the world’s human populations cannot yet be hailed as a success. Shortcomings have been exposed, and these need to be addressed to implement gene-based vaccination programs more effectively. Approaches to pandemic preparedness may take two forms [30]. The first entails building of platforms that may be rapidly adapted and approved for vaccination against new pathogens. Gene-based mRNA synthesis and Ad engineering are examples of these platforms. The second pandemic preparedness strategy involves building stocks of materials that may be used to counter pathogens that are most likely to cause future pandemics. Assembling these resources is based on information about the 30 families of viral pathogens that are most likely to cause pandemics [31]. Material ready for deployment according to this approach may comprise vaccines, antibodies for passive immunization (recombinant protein or nucleic acid-encoded) and antivirals, but the strategy does have risks. Anticipating pathogens that will cause future pandemics is changeable and, as has become clear from the COVID-19 pandemic, viruses may evolve to produce variants with properties that are different to the parental strains.

There are several basic research priorities that are important to improve vaccination development programs. To optimize durability, breadth and strength of humoral and T cell responses, more detailed understanding the basic biology of immunization regimens, ideal dosing and the role of boosters will be fundamental. Vaccines that prevent infection by variants and different viruses from the same family, e.g., by inducing sarbecovirus antibodies, will be beneficial. The report of broad coronavirus neutralization by antibodies that mimic angiotensin converting enzyme 2 (ACE2) receptor is an interesting development [32], and inducing antibodies that mimic ACE2 to counter coronaviruses that engage this receptor will be valuable. Generation of multivalent vaccines, such as may be achieved with virus-like particles comprising antigenic mosaics or the inclusion of several mRNAs encoding different proteins into single LNPs, also show potential beyond COVID-19 [30].

A point, perhaps not made frequently enough, is that the Spike protein of SARS-CoV-2 turned out to be an easy target for vaccine development and was based on years of research on similar Betacoronavirus prototype pathogens [33]. Other viruses often do not present such convenient immunogenic antigens. Repeated failure of HIV-1 vaccine development has shown this. However, demonstration that anti-SARS-CoV-2 mRNA vaccines may be mass-produced for global immunization programs was an important milestone. Extrapolation of current gene-based vaccine technology to counter different infections is an exciting prospect. Also, ensuring that vaccines are robust and affordable will be vital to improve global access. Research on gene therapy and other topics will continue to complement advances with vaccine development. Currently there is significant global resolve to use multifaceted systems to tackle existing problems of pandemic preparedness. The field of gene-based vaccination has impetus and will no doubt witness significant breakthroughs soon.
